# Factors Influencing Burnout Syndrome and Depression in Dentists across Various Institutions

**DOI:** 10.3390/medicina60030517

**Published:** 2024-03-21

**Authors:** Levent Ciğerim, Zeynep Dilan Orhan, İbrahim Doğru, Nazlı Hilal Kahraman, Abdalrahim Hussein, Hayrettin Baş

**Affiliations:** Department of Oral and Maxillofacial Surgery, Faculty of Dentistry, University of Van Yuzuncu Yil, Van 65090, Turkey; zeynepdilanorhan@gmail.com (Z.D.O.); dt.ibrahimdogru@gmail.com (İ.D.); nazliiihilalkahraman@gmail.com (N.H.K.); ab1994d@gmail.com (A.H.); dthayrettinbas@gmail.com (H.B.)

**Keywords:** burnout, depression, dentist, emotional exhaustion, faculty, ODHC, private clinic, dentistry

## Abstract

*Background and Objectives*: This study aimed to identify the occupational and personal factors influencing burnout syndrome (BS) and depression among dentists in academic faculties, oral and dental health centres (ODHCs), and private clinics. *Materials and Methods*: This prospective, cross-sectional study was carried out on dentists working in different regions of Turkey. Data were gathered through an online questionnaire hosted on Google Forms. The questionnaire consisted of demographic data and Maslach BS Inventory (MBI) and Beck Depression Inventory (BDI) sections. The demographic data collected included age, height, weight, marital status, blood type, gender, monthly income, income satisfaction, and whether the participant had enough free time. The dentists were divided into three groups, namely, faculty setting, private clinic, and ODHC, according to the institutions at which they worked. *Results*: The study was composed of 290 dentists, including 172 males and 118 females, with an average age of 36.98 ± 5.56 years. In total, 128 of the dentists worked in faculties, 72 worked in private clinics, and 90 worked in ODHCs. The study found that women exhibited higher EE scores than men (*p* < 0.05). The comparison of BS and depression scores showed no statistically significant differences between groups based on marital status or blood type (*p* > 0.05). There was no significant relationship between emotional exhaustion (EE), depersonalisation (DP), personal accomplishment (PA), and depression scores according to age, BMI, and work experience (*p* < 0.05). It was found that the EE scores of the dentists working in faculties and private clinics were lower than those of the dentists working in ODHCs (*p* < 0.05). Monthly income was associated with depression (r = −0.35). *Conclusions*: The findings reveal that dentists employed in ODHCs reported greater levels of EE. These results suggest a pressing need for enhancements in the work environments of dentists, especially in ODHCs.

## 1. Introduction

Burnout syndrome (BS) results from prolonged exposure to interpersonal stressors in the workplace, often leading to reduced job performance and various types of resignation, including absenteeism and the intention to leave a job [1]. The World Health Organization officially declared BS a disorder following the publication of the 11th revised International Statistical Classification of Diseases and Related Health Problems. It has a negative impact on organisations, clinics, and businesses, resulting in poor patient care, increased workplace absenteeism, clinical mistakes, and financial losses [2,3]. The World Health Organization (WHO) defined it based on the International Classification of Diseases (ICD)-11 as follows: Burnout results from chronic stress that cannot be successfully managed in the workplace and is characterised by three dimensions: (1) loss of energy or a feeling of fatigue; (2) mental detachment from one’s work or negative feelings or pessimism about one’s work; and (3) reduced professional effectiveness. Burnout refers specifically to work-related issues and should not be used to describe experiences in other areas of life [4].

In essence, a lack of resources or a worker’s inability to adapt to job requirements can lead to various forms of resignation, such as absenteeism and even intentions to leave the job. It is associated with problems related not only to one’s work situation but also to other variables, such as learning disabilities, learning theory, one’s social environment, and/or personality factors. In other words, even though they all work in the same environment, there are some workers who suffer from BS and some who do not. One possible mechanism by which the response of employees to a stressful work environment may be differentiated is through personality traits. These may provide a management strategy that enables individuals to obtain or preserve resources and avoid deviance, or they may increase vulnerability and susceptibility to stress factors [5]. Emotional stability was found to be negatively connected to the main elements of BS, depersonalization (DP), and emotional exhaustion (EE), and positively connected to personal accomplishment (PA). BS is less likely in people who are more extraverted and emotionally stable [6]. The question of how BS differs from depression and anxiety, or whether they are complementary, has not been answered. This is important because BS could be diagnosed incorrectly as depression and/or anxiety, resulting in incorrect treatment [7].

Researchers have examined the extent to which individuals who are both burned out and depressed attribute these feelings to their jobs [7,8]. They found that the proportion of people who reported work to be the cause of their BS was proportional to the number of people who reported work to be the cause of their symptoms of depression [9]. An intersection between BS and depression might exist in relation to their causes. Several studies have also shown that BS is linked to depression. Inventories assessing BS, particularly EE, have been found to correlate positively with depressive symptoms [10,11,12,13]. In the literature, there is one study evaluating the relationship between BS and depression levels in dentists, in which the effect of work-related factors was evaluated and it was shown that BS and depression were related. It is not known whether the current findings in dentists are due to BS or depression [8]. However, although BS and depression share some similarities, a number of researchers believe that BS and depression are two different phenomena and that EE has nothing in common with depression [14]. Some studies have shown that there is no intersection between BS and depression [2], stating that, in fact, BS is distinct from depression. In addition, an issue that appears to be important in distinguishing BS from depression is the nature of BS, which is work-related and situation-specific, whereas depression is beyond context and universal. This means that BS tends to be specifically related to a person’s work context, whereas depression may occur regardless of context [9].

If we examine BS independently of depression, the description of work-related BS reveals a range of symptoms, such as physical exhaustion, EE, and loss of motivation, associated with a negative psychological state at work [5]. Other signs of BS have also been identified, including losing touch with family and friends and ignoring personal commitments and hobbies in favour of work. BS has been defined as becoming unmotivated or disengaged, especially when commitment fails to produce the desired results for a purpose or relationship. Constant work can lead to BS among healthcare professionals. BS is a highly negative outcome of chronic work-related stress, with negative personal, business, occupational, and social consequences [12]. It is most often linked to exogenous variables that are unique to a job, though it can also have underlying causes linked to personal characteristics [9,10].

Dentistry is generally associated with high levels of BS [5,15,16,17,18]. As well as care-related stress, there are elements of stress that make dental professionals more susceptible to BS. The stress factors/sources in dentistry are as follows: The work variables include patient overload, working alone, pressure to meet appointment time targets, the requirement to build relationships with confidence and fidelity, and the need to ensure satisfaction with a treatment, as an individual is free to choose their dentist and dental procedure [19]. The behavioural variables include visual, aural, and kinaesthetic challenges, as well as the coordination of fine motor skills. The business variables linked to this occupation include whether a dentist is the owner of a dental practice, is in control of ancillary staff, and/or has variable income. The factors related to personality include the leadership style, emotional competence, and commitment and flexibility of an individual [20]. The other factors are the demographic and economic variables associated with dentistry. As the number of dentists has increased in recent years, the ratio of dentists to the population has decreased. This means the number of dental professionals now outnumbers the population served. This could have an impact on the physical and mental health of dentists, as well as on the standard and number of dentistry services they deliver [16,21].

BS is not a sudden event; it develops in stages, and its severity increases in all three dimensions [3,14]. The first stage is EE, defined as low energy, mental and energy depletion, persistent fatigue, and feelings of powerlessness. Those affected are overcome and unable to cope emotionally. The second stage is DP, defined by being irritable, cynical, moody, dismissive or negative towards the recipients of one’s output, emotionally distant, or lacking empathy. It is a defence mechanism by which people try to prevent their feelings of frustration. The third stage is low PA, defined by low self-esteem, inadequate work skills, persistent poor behaviour, poor self-perception, a sense of failure, withdrawal from social interaction, mood swings, and poor problem-solving skills in both work and personal life [5,6]. The relationship between BS and depression is controversial in the literature [22,23,24]. We believe that it would be insufficient to assess the BS status of dentists only in terms of BS. This is because a dentist who is depressed is likely to give similar answers to questions, and both depression and BS may be present at the same time. Since these two concepts have similar characteristics, we believe that evaluating them together will help to eliminate dentists’ problems or fill in the gaps in their treatments. Therefore, it is important to assess individual characteristics as well as work-related factors when evaluating both BS and depression. As BS is defined as a work-related disorder, we aimed to evaluate the effect of the work institution, which is one of the main work factors leading to BS. However, as the characteristics in the subscales of BS may be confused with depression or BS may be associated with depression, we also aimed to evaluate the levels of BS and depression in individuals and their relationships with each other in terms of various personal characteristics other than the work institution. In this way, we believe that determining the work and individual factors that cause BS and revealing their relationship with depression will play an effective role in the treatment of BS in dentists. The aim of this study was to determine the job-related and individual features that influence burnout syndrome (BS) and depression among dentists.

## 2. Materials and Methods

This observational, cross-sectional study was carried out at the University of Van Yuzuncu Yil Faculty of Dentistry between 15 March and 19 September 2022. Approval for the study was granted by the University of Van Yuzuncu Yil Non-Interventional Clinical Research Ethics Committee (decision number: 2022/03-04). This study was conducted according to the 2013 Declaration of Helsinki.

Active dentists working in a faculty setting, oral and dental health centre (ODHC), or private clinic who had no systemic diseases, took minimal work hours per week, and could take holidays were included in the study. Dentists who were supervisors or employers at their respective institutions, had received psychological support and/or psychiatric treatment in the previous 6 months, had used antidepressants, anxiolytics, sedatives, and/or hypnotics for any reason, and had not fully completed the form were excluded from this study. During the study period, it was found that 23 of the 313 dentists who participated in the study online, hailing from different regions of Turkey, met the inclusion criteria but did not fully complete the forms, and these dentists were also excluded from this study. The dentists were divided into 3 groups, namely, faculty setting, private clinic, and ODHC, according to the institutions at which they worked. The dentists who worked in a faculty setting had 40 h of working time per week, weekends off, no on-call shifts, and a variable number of patients seen per day (between 2 and 10). The dentists who worked at private clinics had 40 h of working time per week, attended to 5–15 patients per day, and worked on Saturdays too. The dentists who worked for ODHCs had one on-call shift per month, but this shift was within their 40 working hours and thus not additional time, and they attended to 20–30 patients per day. As a result, 290 dentists were included in this study, hailing from dentistry faculties of universities, namely, 9 Eylül University, Atatürk University, Hatay Mustafa Kemal University, Karadeniz Technical University, Karamanoğlu Mehmetbey University, Tekirdağ Namık Kemal University, and Van Yüzüncü Yıl University, as well as private clinics and ODHCs in the same cities, i.e., İzmir, Erzurum, Hatay, Trabzon, Karaman, Tekirdağ, and Van.

Data were collected using an online questionnaire created using Google Forms. The link to the survey was shared through social media and phone applications accessible to dentists. Dentists clicked on the link, accessed the survey form, and completed the form. The questionnaire settings allowed for a single entry, preventing the form from being completed again by the same dentist. The questionnaire consisted of demographic data and Maslach BS Inventory (MBI) and Beck Depression Inventory (BDI) sections. The demographics analysed were age, height, weight, marital status, blood type, gender, monthly income, income satisfaction, and whether a participant had enough free time. The MBI consisted of 22 questions with 3 sub-items: emotional BS, DP, and low PA. Each question had a rating on a scale of 1–5, and a total score was calculated. For emotional BS, which consisted of 9 questions, a score of 0–11 indicated low BS, 12–17 indicated moderate BS, and a score greater than 18 indicated high BS. For DP, which consisted of 5 questions, a score of 0–5 indicated low BS, 6–9 indicated moderate BS, and a score greater than 10 indicated high BS. For PA, which consisted of 8 questions, a score greater than 26 indicated low PA, 22–26 indicated moderate PA, and 0–21 indicated high PA [25]. The BDI consisted of 21 questions, and the overall scoring was executed by providing a score between 0 and 3 for each question. When the total score was evaluated, 1–10 was considered normal; 11–16 was considered mild mood disturbance; 17–20 was considered clinically depressed; 21–30 was considered moderately depressed; and 31–63 was considered severely depressed [26].

Using G*Power version 3.1.9.7, the sample size of the study was calculated, amounting to 94 subjects for each group, with α = 0.05, an effect size of 80%, and a power of 90%. It was assumed that at least 284 dentists from 3 groups, i.e., faculty setting, private clinic, and ODHC, would be included in the study.

Statistical analyses were performed using SPSS software version 25. Visual (histogram and probability plot) and analytical (Kolmogorov–Smirnov) methods were used to determine whether the data were normally distributed. Descriptive statistics were used for the demographic and clinical data to determine distribution norms. Comparisons between EE, DP, personal achievement, and depression scores according to gender and marital status were made using the Independent Groups *t*-test when variables met the assumptions of a normal distribution; when data were not normally distributed, the non-parametric equivalent Mann–Whitney U test was used. Comparative analyses of EE, DP, personal achievement, and depression scores according to blood groups and according to institution were examined using one-way ANOVA if the variable in question was normally distributed and the Kruskal–Wallis test otherwise. In cases where the result of the one-way ANOVA test was significant (*p* < 0.05), the Tukey test, a post hoc test, was used to investigate between which two groups this difference originated. Similarly, if the result of the Kruskal–Wallis test was significant, the Mann–Whitney U test was used to determine between which two sets of data this difference originated. Finally, the relationship between monthly income and variables such as EE, DP, PA, and depression was analysed using Pearson’s test when the data were normally distributed and Spearman’s test when they were not. The correlation coefficients were interpreted as follows: 0.05–0.3, low or insignificant; 0.3–0.40, low–moderate; 0.4–0.6, moderate; 0.6–0.7, good; 0.7–0.75, very good; and 0.75–1.00, an excellent correlation. A type 1 error level of 5% was used for statistical significance.

## 3. Results

The demographic characteristics of the dentists are given in Table 1.

Of the 290 dentists, 266 (91.72%) had high EE, 18 (6.21%) had moderate EE, and 6 (2.07%) had low EE. A total of 192 (66.21%) dentists had high DP, 94 (32.41%) dentists had moderate DP, and 4 (1.38%) dentists had low DP. A total of 220 dentists (75.87%) had low levels of personal achievement, 38 dentists (13.10%) had moderate levels of personal achievement, and 32 dentists (11.03%) had high levels of personal achievement. The high rates of EE observed were 90.63% for dentists working in a faculty setting, 83.84% for dentists working in a private clinic, and 100% for dentists working in an ODHC. The rates of high DP were 62.5% for dentists working in a faculty setting, 61.11% for dentists working in a private clinic, and 75.56% for dentists working in an ODHC. It was observed that the rates of low personal achievement were 75% for dentists working in a faculty setting, 83.34% for dentists working in a private clinic, and 71.11% for dentists working in an ODHC (Table 2).

Of the 290 dentists, 30 (10.34%) had severe depression, 106 (36.55%) had moderate depression, 76 (26.21%) had mild depression, and 78 (26.9%) did not have depression. It was observed that 12.5% of the dentists working in a faculty setting had severe depression and 35.94% had moderate depression, while 2.77% of the dentists working in a private clinic had severe depression and 33.34% had moderate depression. Furthermore, 13.33% of the dentists working in an ODHC had severe depression, and 40% had moderate depression. Other distributions are shown in Table 3.

When comparing the scores of EE, DP, PA, and depression by gender, there was only a difference between the groups in terms of EE. It was observed that women’s EE scores were higher than men’s (*p* < 0.05). There were no differences in the scores of DP, PA, and depression according to gender (*p* > 0.05) (Table 4).

When comparing the scores of EE, DP, PA, and depression according to marital status, no statistically significant difference was found between the groups. The scores of EE, DP, PA, and depression of married and single dentists were similar (*p* > 0.05) (Table 5).

No statistically significant differences were found between the scores of EE, DP, PA, and depression according to blood group (*p* > 0.05) (Table 6).

When the EE, DP, PA, and depression scores were compared according to age, BMI, and work experience, no significant relationship was found (*p* < 0.05). On the other hand, there were positive and moderate relationships between EE and depression scores (r = 0.43) and DP and depression scores (r = 0.42) and negative and moderate relationships between PA and depression scores (r = 0.5) (Table 7).

In the intergroup comparisons made to investigate whether scores of EE, DP, PA, and depression differed according to the institution of employment, the only statistically significant difference found was for EE (*p* < 0.05). We found that this difference was caused by both faculty–ODHC and private clinic–ODHC relationships. We also found that the emotional BS scores of the dentists working in faculties and private clinics were lower than those of the dentists working in ODHCs (*p* < 0.05). On the other hand, no difference in EE was found between those working in faculties and private clinics (*p* > 0.05) (Table 8).

When analysing the relationship between monthly income and EE, DP, PA, and depression scores, there was only a relationship with depression, and this relationship was negative and of low to moderate significance (r = −0.35). On the other hand, no significant relationship was found between monthly income and EE, DP, and PA scores (r < 0.3) (Table 9).

## 4. Discussion

Burnout syndrome has become increasingly prevalent across professions since the COVID-19 pandemic, significantly affecting health professionals, including dentists [2]. Dentists are constantly in contact with people for professional reasons. Apart from patient-related factors, occupational, individual, and other external factors affect the BS levels of dentists. Assessment of BS reveals that high EE is often associated with the syndrome, and this study’s findings corroborate that EE is notably prevalent among dentists [6]. For this reason, in this study, we used the parameter of high EE as a reference when discussing BS. While the rate of EE in dentists was 0.9% in a study conducted in 2008 in Turkey, it was shown that this rate was 38% in a study conducted in 2016 and 73.5% in a study conducted in 2023 [8,27,28]. In this study, this rate was found to be 91.72%. In a study conducted in Egypt in 2022, it was reported that the rate of emotional BS was 62% [29]; in a study conducted in Spain in the same year, this rate was 61.3% [5]; and in a study conducted in Peru in 2020, it was 98.09% [18]. When these results were analysed, a dramatic increase in the prevalence of BS syndrome among dentists was observed from the past to the present, not only in Turkey but also in many other countries. The impact of COVID-19 on this increase is an undeniable fact when considered broadly on a country-by-country basis [30]. As dentists working in different regions of Turkey were included in this study, it would be appropriate to interpret our results for Turkey in general. The present study found that BS was higher among dentists working in ODHCs than among those working in faculties and private clinics. The BS levels of dentists working in faculties and private clinics were similar. These results support the findings reported by Gürses et al., who stated that the reason for this result was the high number of patients in ODHCs [28]. We agree with this conclusion and also believe that the use of shifts and night shifts in ODHCs, shifts not available in other institutions, is another reason for this result. Unlike dentists working in other facilities during the pandemic, the majority of dentists working in ODHCs were actively involved in COVID-19 field work rather than providing routine dental services. They performed their duties in different working conditions to which they were not accustomed and with personal protective equipment that was difficult to adapt. We believe that these additional reasons have caused the BS rate of dentists in ODCHs to be higher. Clearly, the reasons we have mentioned are a source of stress, and as a result, they cause BS among dentists. Apart from the unhappiness caused by these work-related stress factors, we think that dentists’ EE increases when their expectations are not met and when they are disappointed due to insufficient monthly earnings. This study found that there was a relationship between monthly income and depression levels, even at low levels. This finding supports the view that depression may also contribute to the high levels of BS among dentists with low monthly incomes and that work-related stressors may also lead to depression.

In the literature, analysing the relationship between BS and gender revealed that EE was significantly higher in female dentists, consistent with findings from previous studies [8,17] and this study. In addition, their DP, PA, and depression levels were similar to men. It is posited that the heightened level of emotional burnout among female dentists may stem from the fact that women are often more affected by work-related stress factors and physical fatigue. It is also possible that female dentists who are unhappy at work are more likely to express their discomfort and feelings emotionally compared to men. In their study, Mocny-Pachońska et al. found that the stress levels of female dental students were higher than those of males [31]. Queirolo et al. reported that patient confrontation caused more anxiety among female dentists [32]. These findings support our thoughts about higher burnout among female dentists. In this study, there were no differences in BS and depression levels according to marital status, age, BMI, work experience, or blood group. The fact that there were no differences between the groups according to these factors, which we can consider single factors, i.e., the effects of the single factors were similar, suggests that the high levels of BS and depression among dentists in the current study were due to the institutions in which they worked. In this study, marital status was found to have no effect on burnout, supporting the findings of Huri et al. and Radwan and Mursy [8,29]. Gómez-Polo et al., Jin et al., and Kanai-Pak et al. found a relationship between age and burnout and reported that younger people had higher levels of burnout [5,33,34]. In this study, contrary to the literature, no relationship was found between age and burnout. We believe that the main reason for this difference is the low average age of the dentists included in this study and the low number of older dentists included. Bahlaq et al. found that the BMI levels of dental and medical students were associated with burnout [35]. Vasquez-Purí et al. reported that BMI had no effect on the burnout levels of health professionals working in public hospitals [36]. This study found that the BMI of dentists was not associated with burnout. Slabšinskienė et al., Gorter et al., and Gómez-Polo et al. reported that work experience and BMI were related and that burnout was higher among dentists with less work experience [5,15,17]. In this study, contrary to the literature, no relationship was found between work experience and burnout. We believe that this difference is due to the fact that the mean work experience of the dentists included in the study was lower than in other studies. We did not find any studies in the literature that investigated the relationship between the blood groups of either dentists or healthcare workers and burnout. In this study, no relationship was found between the blood groups of dentists and their burnout levels. Yadav et al. showed that the most common blood group among dental students was group B [37]. In this study, the most common blood group among dentists was group A.

There is some debate about whether BS and depression are two different disorders. The fact that emotional BS can be caused by both disorders is the basis of this debate [12,13,38]. To answer this question of whether emotional BS is caused by BS syndrome or depression, we thought that both BS and depression among dentists should be assessed at the same time, so we assessed them both accordingly in this study. In their study investigating BS and depression in dentists, Huri et al. found that BS subscales, especially emotional BS, were strongly correlated with depressive outcomes [8]. In this study, emotional BS, DP, and low PA among dentists were found to be correlated with depression levels, supporting the results of the study by Huri et al. In this study, the EE levels of dentists working in OHDCs were found to be higher than those working in other institutions, and the reason for this is the number of patients seen during the day and the types of shifts in OHDCs not used by other institutions. As all three institutions require dentists to work 40 hours per week, it is obvious that the main difference is in the number of patients seen. While dentists working in faculties and private clinics see an average of 10 patients per day, this number is 25 in ODHCs. The finding of high BS levels among dentists in the present study indicates that improvements should be made in terms of working hours, shifts, number of patients seen, and monthly income, which are workplace-related factors. Reducing the number of patients seen, increasing the amount of time spent with patients, and revising the monthly income according to the current economic conditions are among the first tasks required to lower BS. In order to make these arrangements, it is necessary to give priority to this issue in health policy and planning. Otherwise, further deterioration of the current health status of dentists in terms of burnout will become a situation that will be difficult to manage, to the detriment of society. The correlation between BS and depression levels found in this study suggests that, if we accept that BS syndrome reflects work-related BS, then work-related BS leads to depression, or depression prevents people from coping with work-related stressors. In either case, it is obvious that dentists will show similar results. The limitations of this study are as follows: The data in this study consist of responses to the questionnaires given at one time. If the evaluation had been carried out at two or more different times, the results might have been different. The fact that the dentists answered the questions at different times of the day, i.e., before, during, or after work, may have influenced the results because it may have affected their current emotional states. Potential differences in the dentists’ current psychological, emotional, and health statuses may have influenced the results, along with the presence of unrecognised BS and/or depression.

## 5. Conclusions

In summary, the study found that dentists employed in ODHCs experienced higher emotional exhaustion (EE), and it was observed that dentists in general had higher levels of BS. The analysis also revealed a correlation between dentists’ monthly income and their levels of depression, suggesting that financial factors contribute to the incidence of BS and depression. These results indicate a pressing need for improvements in the working conditions for dentists, particularly in ODHCs, to mitigate the factors contributing to burnout. It is necessary to plan health policies that include improving the number of patients in institutions, working hours, shifts and monthly earnings. We think that in the treatment of dentists suffering from burnout, taking the presence of an existing depression into account as well as the predisposing factors is important for the success.

## Figures and Tables

**Table 1:** The demographic characteristics of the dentists.

		Mean ± SD	Min–Max
Age	26.98 ± 5.6		21–49
BMI	23.52 ± 3.6		16.652–34.602
Work Experience (year)	4.72 ± 4.5		0-25
		N	%
Gender	Male	172	59.3
	Female	118	40.7
Marital Status	Married	82	28.3
	Single	208	71.7
Institution	Faculty	128	44.2
	Private Clinic	72	24.8
	ODHC	90	31
Income	$500–1000	144	49.65
	$1000–1500	82	28.27
	$1500–2000	42	14.48
	$2000 and over	22	7.58
Income Satisfaction	Sufficient	16	5.52
	Partly Sufficient	102	35.17
	Insufficient	172	59.31
Enough Free time	Yes	56	19.32
	No	234	80.68
Blood Group	A	102	35.2
	B	56	19.3
	AB	26	9.0
	0	76	26.2
	Unknown	30	10.3

BMI: body mass index; SD: standard deviation.

**Table 2:** Distribution of BS levels according to institution.

	Emotional Exhaustion			Depersonalisation			Personal Accomplishment		
	Low	Moderate	High	Low	Moderate	High	Low	Moderate	High
Faculty	4 (3.12 ^a^) (66.67 ^b^)	8 (6.25 ^a^) (44.45 ^b^)	116 (90.63 ^a^) (43.60 ^b^)	4 (3.12 ^a^) (100 ^b^)	44 (34.38 ^a^) (46.81 ^b^)	80 (62.5 ^a^) (41.67 ^b^)	96 (75 ^a^) (43.64 ^b^)	14 (10.94 ^a^) (36.84 ^b^)	18 (14.06 ^a^) (56.25 ^b^)
Private Clinic	2 (2.77 ^a^) (33.33 ^b^)	10 (13.89 ^a^) (55.55 ^b^)	60 (83.34 ^a^) (22.56 ^b^)	0 (0 ^a,b^)	28 (38.89 ^a^) (29.79 ^b^)	44 (61.11 ^a^) (22.91 ^b^)	60 (83.34 ^a^) (27.27 ^b^)	6 (8.33 ^a^) (15.79 ^b^)	6 (8.33 ^a^) (18.75 ^b^)
ODHC	0 (0 ^a,b^)	0 (0 ^a,b^)	90 (100 ^a^) (33.84 ^b^)	0 (0 ^a,b^)	22 (24.44 ^a^) (23.40 ^b^)	68 (75.56 ^a^) (35.42 ^b^)	64 (71.11 ^a^) (29.09 ^b^)	18 (20 ^a^) (47.37 ^b^)	8 (8.89 ^a^) (25 ^b^)
Total	6 (2.07 ^a^)	18 (6.21 ^a^)	266 (91.72 ^a^)	4 (1.38 ^a^)	94 (32.41 ^a^)	192 (66.21 ^a^)	220 (75.87 ^a^)	38 (13.10 ^a^)	32 (11.03 ^a^)

^a^: intragroup distribution rate (%); ^b^: intergroup distribution rate (%).

**Table 3:** Distribution of depression levels according to institution.

	Depression Level			
	No	Mild	Moderate	Severe
Faculty	34 (26.56 ^a^) (43.59 ^b^)	32 (25 ^a^) (42.11 ^b^)	46 (35.94 ^a^) (43.4 ^b^)	16 (12.5 ^a^) (53.34 ^b^)
Private Clinic	30 (41.67 ^a^) (38.46 ^b^)	16 (22.22 ^a^) (21.05 ^b^)	24 (33.34 ^a^) (22.64 ^b^)	2 (2.77 ^a^) (6.66 ^b^)
ODHC	14 (15.56 ^a^) (17.95 ^b^)	28 (31.11 ^a^) (36.84 ^b^)	36 (40 ^a^) (33.96 ^b^)	12 (13.33 ^a^) (40 ^b^)
Total	78 (26.9 ^a^)	76 (26.21 ^a^)	106 (36.55 ^a^)	30 (10.34 ^a^)

^a^: intragroup distribution rate (%); ^b^: intergroup distribution rate (%). ODHC: oral and dental health centre.

**Table 4:** Comparison of EE, DP, personal achievement, and depression scores according to gender.

	Gender		
	Male (Mean ± SD)	Female (Mean ± SD)	*p*
EE	26.87 ± 7.7	30.3 ± 8.5	0.013 *
DP	11.93 ± 3.7	12.52 ± 5.4	0.94 **
PA	28.55 ± 4.8	28.72 ± 5.4	0.928 **
Depression	16.2 ± 11.54	17.76 ± 12.9	0.501 **

SD: standard deviation; EE: emotional exhaustion; DP: depersonalisation; PA: personal accomplishment. * Independent Groups *t*-test; ** Mann-Whitney U Test; *p* < 0.05.

**Table 5:** Comparison of EE, DP, personal achievement, and depression scores according to marital status.

	Marrital Status		
	Married (Mean ± SD)	Single (Mean ± SD)	*p*
EE	28.53 ± 9.3	28.16 ± 7.7	0.806 *
DP	11.9 ± 5.1	12.3 ± 4.3	0.368 **
PA	38.34 ± 5.2	28.74±	0.881 **
Depression	15.6 ± 11.4	17.3 ± 12.4	0.339 **

SD: standard deviation; EE: emotional exhaustion; DP: depersonalisation; PA: personal accomplishment. * Independent Groups *t*-test; ** Mann-Whitney U Test; *p* < 0.05.

**Table 6:** Comparison of EE, DP, personal achievement, and depression scores according to blood group.

	A (Mean ± SD)	B (Mean ± SD)	AB (Mean ± SD)	0 (Mean ± SD)	Unknown (Mean ± SD)	*p*
EE	28.5 ± 8.3	39.5 ± 7.2	26 ± 7.4	27.8 ± 8.8	26.4 ± 8.6	0.41 *
DP	12.98 ± 4.7	12.8 ± 4.3	10.4 ± 2.4	11.6 ± 5.4	11.5 ± 2.5	0.155 **
PA	28.0 ± 5.4	29.1 ± 4.3	25.7 ± 6.0	30.1 ± 3.6	28.6 ± 6.3	0.132 **
Depression	19.1 ± 13.9	17.9 ± 12.9	19.3 ± 7.3	13.8 ± 10.6	12.7 ± 9.2	0.15 **

SD: standard deviation; EE: emotional exhaustion; DP: depersonalisation; PA: personal accomplishment. * one-way ANOVA; ** Kruskal–Wallis Test; *p* < 0.05.

**Table 7:** Evaluation of the relationship between age, BMI, work experience, EE, DP, personal achievement, and depression variables.

	EE	DP	PA	Depression
Age	r = 0.025 *	r = −0.057 *	r = 0.036 *	r = −0.165 *
Work Experience	r = −0.009 *	r = −0.082 *	r = 0.082 *	r = −0.071 *
BMI	r = 0.043 **	r = 0.057 *	r = 0.058 *	r = 0.023 *
EE	-	-	-	r = 0.43 *
DP	-	-	-	r = 0.42 *
PA				r = −0.5 *

EE: emotional exhaustion; DP: depersonalisation; PA: personal accomplishment; BMI: body mass index. * Spearman Test; ** Pearson Test.

**Table 8:** Comparison of EE, DP, personal achievement, and depression variables according to institution.

	Faculty (Mean ± SD)	Private Clinic (Mean ± SD)	ODHC (Mean ± SD)	*p*
EE	26.6 ± 8.0	26.8 ± 8.4	31.9 ± 7.3	0.001 *
DP	11.6 ± 4.3	11.8 ± 4.1	13.3 ± 5.0	0.191 **
PA	28.2 ± 5.5	29.6 ± 4.9	28.5 ± 4.4	0.545 **
Depression	17.7 ± 14.3	13.3 ± 9.8	18.4 ± 9.8	0.079 **

SD: standard deviation; EE: emotional exhaustion; DP: depersonalisation; PA: personal accomplishment; ODHC: oral and dental health centre. * one-way ANOVA; ** Kruskal–Wallis Test.

**Table 9:** Evaluation of the relationship between monthly income and EE, DP, personal achievement, and depression variables.

	EE	DP	PA	Depression
Monthly Income	r = 0.054	r = −0.013	r = 0.121	r = −0.35 *

EE: emotional exhaustion; DP: depersonalisation; PA: personal accomplishment. * Spearman Test.

## Data Availability

The dataset used in this study is available on request. The data are not publicly available as they contain information that could compromise the privacy of research participants.
